# Phylogeography and evolutionary analysis of African Rotavirus a genotype G12 reveals district genetic diversification within lineage III

**DOI:** 10.1016/j.heliyon.2019.e02680

**Published:** 2019-10-21

**Authors:** Babatunde Olanrewaju Motayo, Olukunle Oluwapamilerin Oluwasemowo, Babatunde Adebiyi Olusola, Adewale Victor Opayele, Adedayo Omotayo Faneye

**Affiliations:** a Department of Virology, College of Medicine, University of Ibadan, Nigeria; b Department of Medical Microbiology, Federal Medical Center, Abeokuta, Nigeria

**Keywords:** Bioinformatics, Virology, Microbial genomics, Epidemiology, Rotavirus, Virus evolution, Phylogeny, Africa

## Abstract

Group A rotavirus (RVA) genotype G12 has spread globally and has become one of the most prevalent genotypes of rotavirus in Africa. To understand the drivers for its genetic diversity and rapid spread we investigated the Bayesian phylogeography, viral evolution and population demography of Rotavirus G12 in Africa. We downloaded and aligned VP7 gene sequences of Rotavirus genotype G12, from thirteen African countries (n = 96). Phylogenetic analysis, Evolutionary analysis and Bayesian Phylogeography was carried out, using MEGA Vs 6, BEAST, and SPREAD3. Phylogenetic analysis revealed that all the African sequences fell into lineage III diversifying into two major clades. The evolutionary rate of the African rotavirus G12 sequences was 1.678×10^−3^, (95% HPD, 1.201×10^−3^ - 2.198×10^−3^) substitutions/site/year, with TMRC of 16.8 years. The Maximum clade credibility (MCC) tree clustered into three lineages (II, III, IV), African strains fell within lineage III, and diversified into three clusters. Phylogeography suggested that South Africa seemed to be the epicentre of dispersal of the genotype. The demographic history of the G12 viruses revealed a steady increase between the years1998–2007, followed by a sharp decrease in effective population size between the years 2008–2011. We have shown the potential for genetic diversification of Rotavirus genotype G12 in Africa. We recommend the adoption of Molecular surveillance across Africa to further control spread and diversification of Rotavirus.

## 1 Introduction

Group A rotavirus has been established to be the main agent responsible for acute gastroenteritis (AGE) among children and infants worldwide. In 2016, it was reported to be responsible for about 128,000 deaths with over 2/3 of cases occurring in sub-Saharan Africa [1]. There are two life attenuated rotavirus vaccines, Rotarix and Rotateq, which have been licensed for use in many countries after large phase 3 clinical trials were conducted in 2006 [2,3]. In 2009 the world health organisation (WHO), recommended the global use of the 2 live attenuated vaccines Rotarix and Rotateq. In Africa, several countries have included rotavirus vaccination in their (EPI) programs [4].

Rotavirus belongs to the virus family Reoviridae, it is a non-enveloped and has an icosahedral nucleocapsid structure, enclosing a double stranded RNA genome segmented into 11 compartments. The rotavirus RNA genome codes for six structural proteins, (VP1 to VP4, VP6 and VP7) and five/six nonstructural proteins (NSP1 to NSP5/6) [5]. There are at least 10 distinct species/groups (A- I, J), differentiated by their VP6 antigenic properties [6]. There are 32 G (VP7) genotypes and 47 P (VP4) genotypes identified through molecular epidemiologyupdate of the Rega Institute, KU Leuven, Belgium https://rega.kuleuven.be/cev/viralmetagenomics/virus-classification/7th-RCWG-meeting.

Molecular epidemiology has identified the widespread circulation of various genotypes of rotavirus in Africa, the globally prevalent genotypes G1P [8], G3P [4] have been reported in various African countries [7, 8, 9, 10, 11]. However these globally prevalent genotypes are being replaced by more recent genotypes such as G9P [8], G8P [4], G12P [6], G12P [8], as previously reported [12]. There have also been recent reports of widespread outbreaks in parts of West Africa such as Nigeria attributed to RVA G12 strains [12, 13, 14]. Report has also suggested the rapid evolution of RVA genotype G12 as a panacea for its global spread [15]. The rapid emergence of this genotype in Africa has not been well understood, however mechanisms such as interspecies recombinations, RNA polymerase infidelity and accumulated point mutations have been attributed to rotavirus evolution [5, 16, 17, 18]. In order to answer some of the questions arising from the rapid spread of RVA genotype G12 in Africa, this study was designed to investigate the Bayesian phylogeny and evolutionary dynamics of RVA genotype G12 strains in Africa.

## 2 Methods

### 2.1 Dataset

A total of 96, 630 nucleotide partial VP7 genotype G12 gene sequences spanning a period of 1998–2013 from thirteen African countries (8 Nigerian, 11 Cameroonian, 4 from Togo, 12 from Democratic republic of Congo, 3 from Kenya, 12 from Mozambique, 2 from Ethiopia, 4 from Malawi, 3 from Uganda, 17 from Burkina Fasso, 14 from South Africa, 5 from Zambia, and 1 from Egypt), were downloaded from GenBank with the help of the Rotavirus resource database. Other information collected were the date and location of isolation, any sequence without these information was not included in the dataset. Along with these were seven reference VP7 rotavirus genotype G12 genome sequences from Argentina, Thailand, Korea, and Japan. The total number of sequences in the dataset was 103. Duplicate sequences with identical year and location were excluded from the dataset.

### 2.2 Phylogenetic analysis

Sequence data was edited with Bioedit http://www.mbio.ncsu.edu/BioEdit/bioedit.html, multiple sequence alignment was done using CLUSTALW version 2.1 software using default settings http://www.uebi.ac.uk/clustalw/. Phylogenetic trees were constructed in MEGA version 6.0 software using Maximum likelihood method with p distance model and 1000 bootstrap replicates www.megasoftware.net.

### 2.3 Evolutionary rate, time scaled phylogeny and population dynamic analysis

Evolutionary and additional phylogenetic analysis was carried out on African Rotavirus genotype G12 strains, using a Bayesian evolutionary approach using Markov Chain Monte Carlo (MCMC) implemented in BEAST version 1.10.4 [19]. Cluster analysis and sequence similarity was carried out using the online tool CD-HIT version 4.6 http://weizhongli-lab.org/cdhit_suite/cgi-bin/index.cgi?cmd=cd-hit-est. The strength of the temporal clock signal of the dataset was carried out by conducting a regression of root to tip genetic distances against year of sampling analysis using TempEst v 1.5 [20]. For the MCMC run, a total of 103 rotavirus virus partial G12 sequences of 630 nucleotides in length were aligned, consisting 96 African sequences and 7 reference sequences from Argentina, Japan Thailand and Korea. A list of the sequences used for the analysis is contained in supplementary table 1. Two clock models were initially evaluated strict and relaxed molecular clock, with four different tree priors, constant population size, exponential population size, Bayesian Skyride plot and Gausian Markov Random Field Skyride plot. Each selected model was run for an initial 30, 000, 000 states. Models were then compare using the Bayes factor with marginal likelihood estimated using the path sampling and stepping stone methods implemented in BEAST version 1.10.4 [19]. Further analysis was then done using the relaxed clock with Gausian Markov Random Field Skyride plot coalescent prior. The MCMC run was set at 100, 000, 000 states with a 10% burn in. Results were visualised in Tracer version 1.8. (http://tree.bio.ed.ac.uk/software/tracer/). The effective sampling size (ESS) was calculated for each parameter, all ESS values were >200 indicating sufficient sampling. Bayesian skyride analysis was carried out to visualise the epidemic evolutionary history using Tracer v 1.8. (http://tree.bio.ed.ac.uk/software/tracer/). The maximum clade credibility tree was selected from the posterior tree distribution after 10% burn-in using TreeAnnotator v 1.8. (http://beast.bio.ed.ac.uk/TreeAnnotator/) and a time scaled MCC tree was visualised in FigTree vs 1.4.

### 2.4 Phylogeographical analysis

Geographical coordinates of the locations of the African rotavirus G12 sequences were retrieved from the web with the help of online servers. A phylogeographic tree with discrete traits was constructed using the African rotavirus G12 sequences and their geographic coordinates in latitude and longitude using a Bayesian stochastic search variable selection (BSSVS) model implemented in BEAST [19]. The discrete trait model allows for incorporation of ecological data with evolutionary analysis, it also acts as a probabilistic substitution model between discrete categories (for instance in our analysis host or location) [21, 22]. The clock prior used for the African G12 sequences were the same as the one used for the population demography. The resulting tree was annotated in TreeAnnotator after discarding a 10% burn-in, and visualised in Figtree. The resulting tree was spatially projected and converted to a Java script object file (JSON) and rendered into the software SPREAD3 [23] after which it was visualised in Hyper-text Markup Language (HTML) format using the SPREAD3 software.

## 3 Results and discussion

**Fig. 1:**
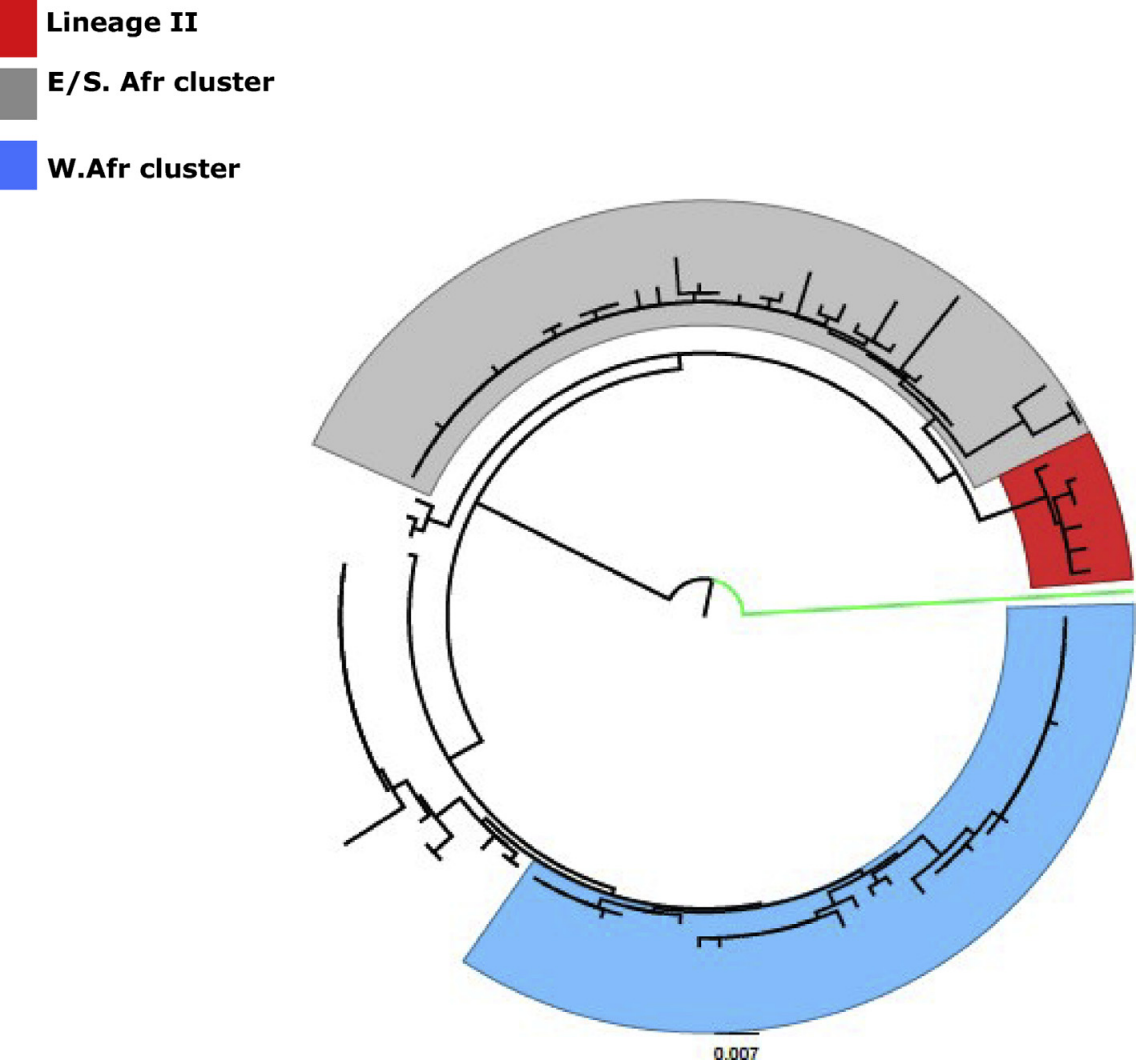
Phylogenetic tree of Rotavirus genotype G12 partial VP7 gene sequences. Tree was constructed using the Maximum likelihood algorithm, with 1000 bootstrap replicates using MEGA version 6.0 software and visualised in Figtree. The West African cluster is highlighted in sky blue, the East/South African cluster highlighted in grey. Lineage 2 reference isolates are indicated in Red, while the lineage IV Porcine isolate is indicated in bright green. Scale bar is indicates number of substitutions per site.

The current study reports the evolutionary dynamics and phylogeoraphy of rotavirus genotype G12 in Africa. Rotavirus genotype G12 has been reported globally, it is reported that a single lineage of this genotype is responsible for its rapid global spread [15]. In this study, 96 partial RVA genotype G12 sequences from thirteen African countries were analysed along with 7 global reference sequences. Cluster analysis using CD-HIT resulted into six different similarity clusters ranging from between 97.62% to 100% amino acid similarity. Phylogenetic analysis of the sequences revealed that all the African sequences fell into lineage III diversifying into two major clades, the West African clade highlighted in sky blue, and the East/South African clade highlighted in grey. Lineage 2 reference isolates are indicated in Red, while the lineage IV Porcine isolate is indicated in bright green (Fig. 1). Study results show that the African rotavirus G12 isolates have evolved into two sub lineages defined largely by geographical location. The reason for this genetic diversification has not been well defined, although factors such as natural boundaries to mass migration of human population such as the high mountain ranges of Central and East Africa, as well as the Sahara desert could serve as major factors. A similar observation of geographically bound genetic diversification was reported in a recent study of Lassa fever virus in Nigeria 2018 [24], where genetic diversification was restricted by rivers which acted as barriers to the cross migration of rodent reservoirs of the virus. In addition, all sequences in our data set had a positive correlation coefficient between genetic divergence and time, supporting the use of Bayesian phylogenetic analysis (Supplementary Figure 1).

**Fig. 2:**
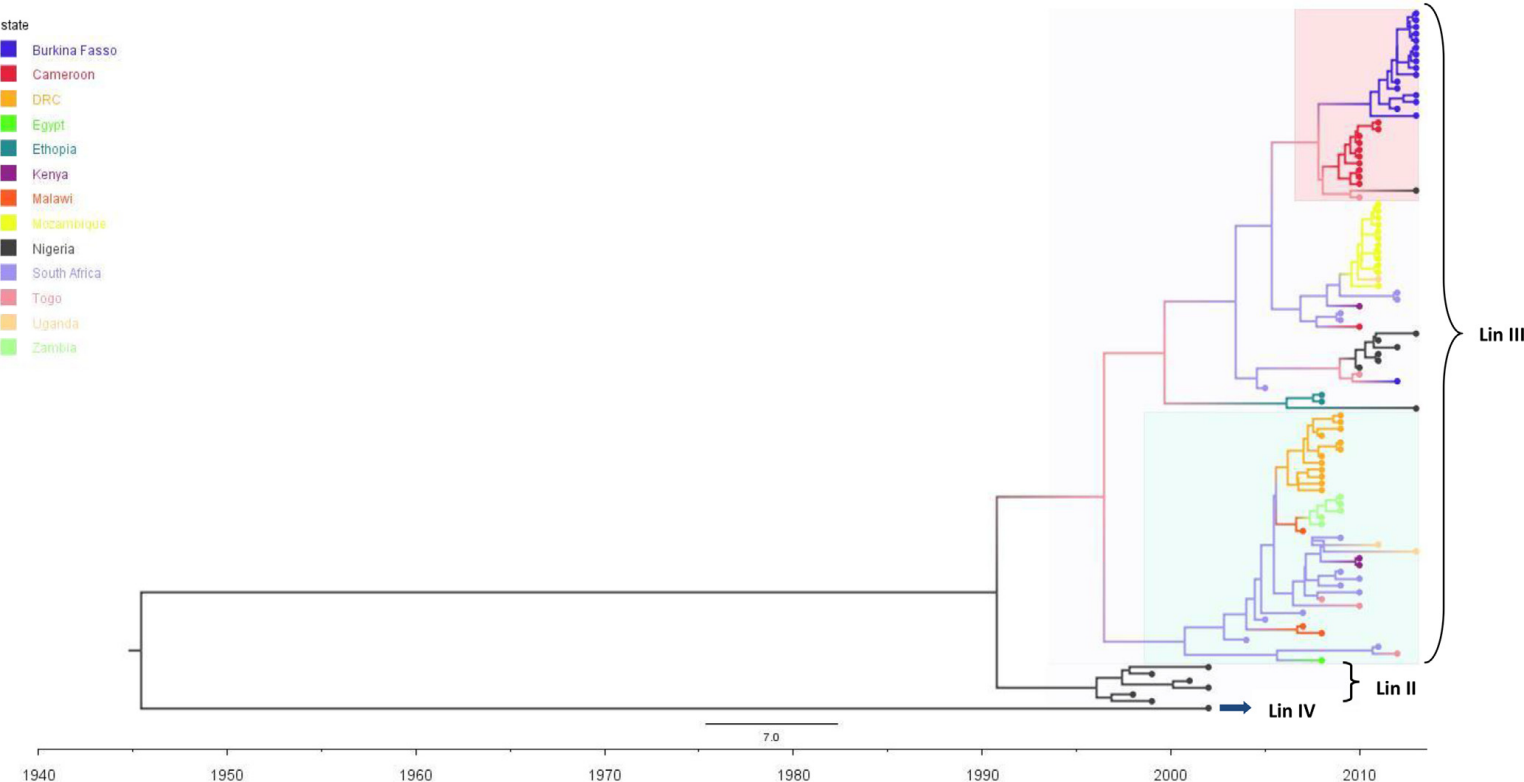
Time scaled Bayesian MCC tree of African Rotavirus virus VP7 genotype G12 sequences. Nigerian strains are indicated in Black, along with the reference G12 strains from Korea, Japan and the Porcine strain. Burkina Fasso strains are indicated in Navy blue, Cameroonian strains are indicated in red, Democratic Republic of Congo are indicated in Orange, Zambian strains are in Light green. South African strains are indicated in Sky blue, Mozambique are indicated in Yellow, Kenyan strains are indicated in Purple. Ugandan strains are indicated in Light orange, while Togolese strains are indicated in Light purple. The reference porcine strain is indicated by blue arrow. The faint Pink horizontal box represents the West African clade, while the faint Blue horizontal box represents the South African clade, within lineage III. Lineages are indicated by horizontal brackets.

Majority of RNA viruses have been reported to have an evolutionary rate of between 1.0×10^−3^ to 1.0×10^−6^ nucleotide substitutions/site/year (subs/site/year) [25]. The evolutionary rate of the analysed African rotavirus G12 sequences was estimated to be 1.678×10^−3^, with 95% high posterior density interval (HPD) of 1.201×10^−3^ to 2.198×10^−3^ subs/site/year. This is slightly lower than the evolutionary rate of 1.89×10^−3^ of rotavirus genotype G9 reported in a global study of rotavirus molecular evolution in 2010 [15]. The evolutionary rate observed in our study hovers around the global rate previously reported for RVA G12 in 2010 [15], this shows that the African strains have a steady mutation rate but possess the potential to diversify rapidly through molecular evolution. The time to most recent common ancestor (TMRC), was calculated to be 16.8 years, dating back to around mid-1996, Table 1 shows the TMRC acording to clusuter and sampling interval. The time scaled MCC tree topology clustered into three lineages (II, III, IV), with all the African trains falling into lineage III. The African G12 strains further diversified into two main sub-clades within lineage III, the West African and South African clusters as shown in Fig. 2. The porcine G12 isolate was the only lineage IV strain, while the reference strains from Argentina, Thailand and Korea fell into lineage II. From the MCC tree the diversification of the 3 clusters within the African lineage III isolates occurred around the same time, between the year 2003 and 2004. The MCC tree topology follows a similar trend with the neighbour phylogeny shown in Fig. 1, further buttressing the earlier mentioned observation of geographically bound diversification of African rotavirus genotype G12.

**Table 1:** Sumary of the study sequences according to sampling interval and most recent common ancestor.

Clade/Cluster	Country (N)	Sampling Interval	TMRC (HPD)
West Africa 1	Burkina Fasso (16), Cameroon (10)	2010–2013	2007, (2004–2009)
South Africa	Mozambique (12), South Africa (4), Uganda (1), Kenya (1), Cameroon (1)	2009–2011	2006, (2001–2008)
West Africa 2	Nigeria (6) Togo (1), Burkina Fasso (1)	2010–2013	2009, (2006–2011)
East Africa	Democratic republic of Congo (12), Zambia (4), Kenya (2), Malawi (3) South Africa (9), Togo (2), Uganda (1), Egypt (1)	2004–2013	2002, (2000–2004)

Key: TMRC = Time to most recent common ancestor, HPD = Highest posterior density, N.B: The table has summarized the study sequences according to the major clusters with Rotavirus G12 lineage III (Fig. 2).

**Fig. 3:**
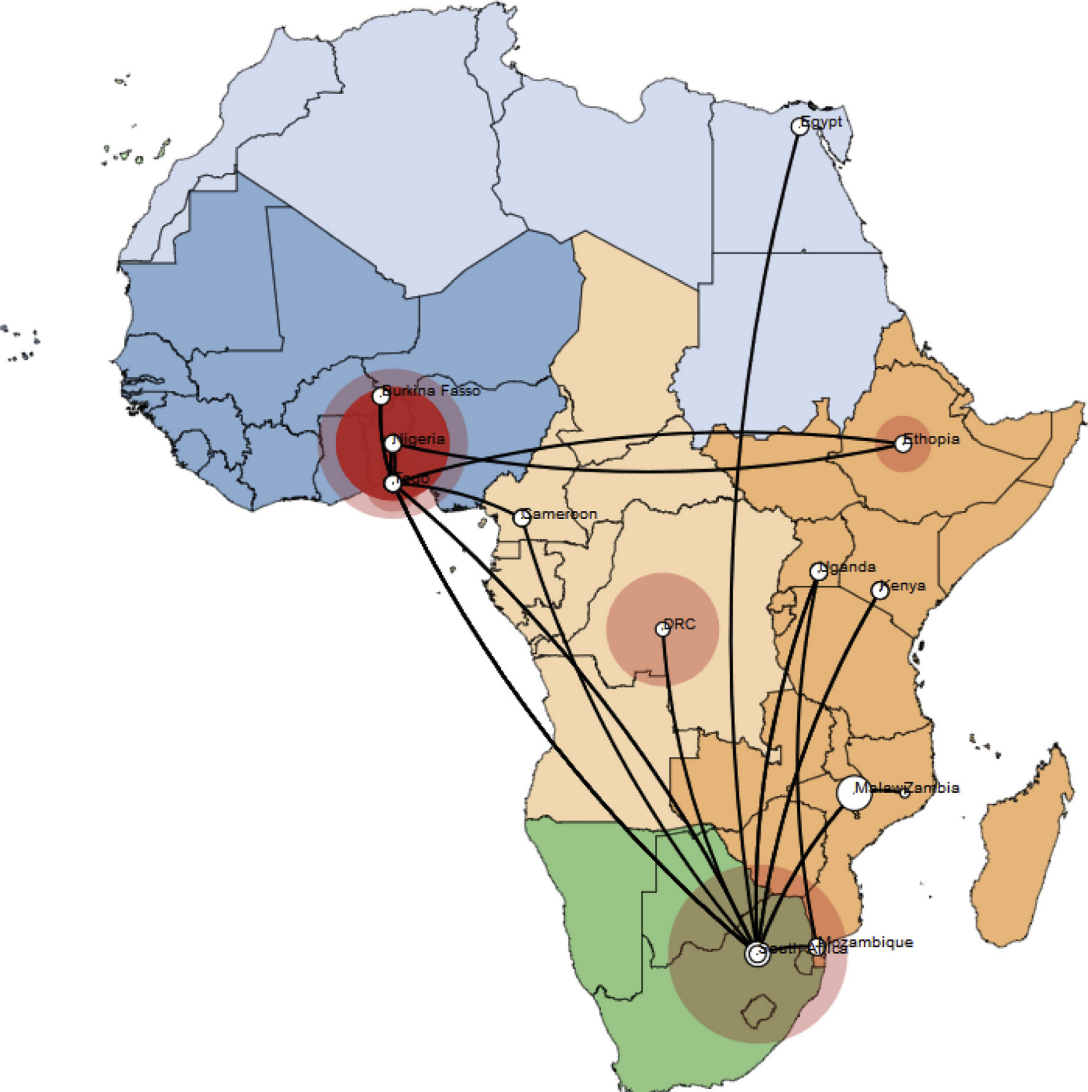
Map showing spatiotemporal viral diffusion of rotavirus genotype G12 across Africa. The map is coloured according to geographical regions. Names of the countries of isolation are written in the map. The size of the circles in each country is proportional to the number of sequences analysed for that country.

Phylogeography showed RVA G12 dispersal from West Africa down to South Africa (Fig. 3), before moving northward through Malawi and Ethiopia before getting to Egypt. From this analysis rotavirus G12 was rapidly dispersed between countries covering long distances within a short period of time. Previous studies have shown the contribution of international travel driven by trade and economic migration to the dispersal of infectious agents across long distance countries [26]. Asides from the long distance dispersal, the genotype was also dispersed from Nigeria, across to neighbouring West African countries of Togo, Cameroon and Burkina Fasso, showing that rotavirus genotypes are capable of both short distance and long distance spread. South Africa was identified to play a major role in the dispersal of the genotype to other regions of the continent. This might not portray the actual picture because of poor molecular surveillance in most parts of Africa [27], but gives a clue to the viral dispersal events within the time frame (1998–2013). However, there have been reports of several RVA G12 outbreaks from southern Africa [27, 28, 29]. A major limitation to this study was the limited number of African RVA G12 sequences available in GenBank during the time frame of data collection (1998–2013).

**Fig. 4:**
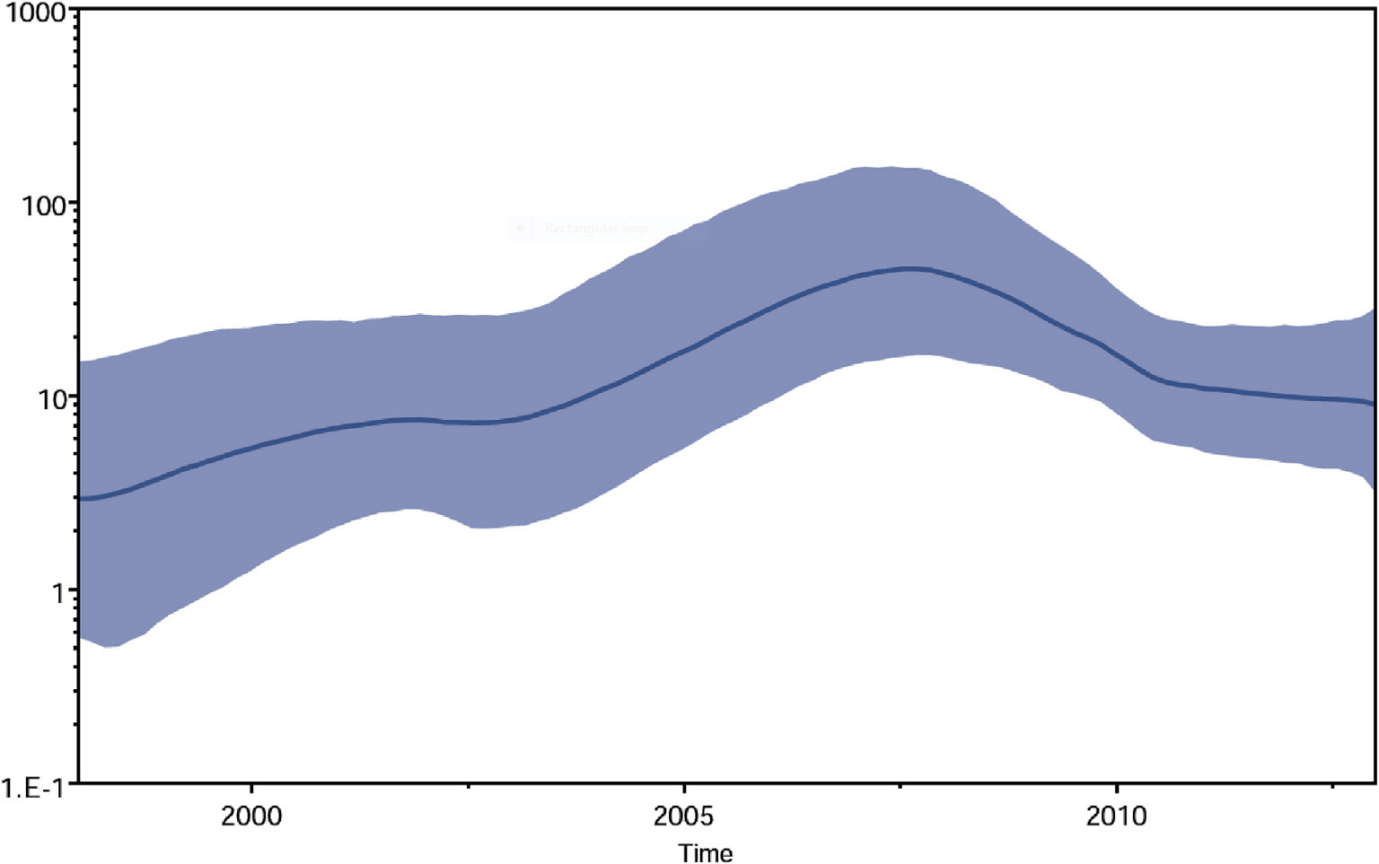
Bayesian skyplot reconstruction of African Rotavirus genotype G12strains, showing the median exponential growth line, with the blue solid area representing the 95% HPD for the growth.

The demographic history of the African G12 viruses estimated through the BSP model, suggested that the genotype experienced a steady increase in its population size (effective number of infections) between 1998 and 2007, and a sharp decrease in virus population between the year 2008–2011 (Fig. 4).

## 4 Conclusions

We have shown that RVA G12 has diversified based on geographical locations. The tendency for further diversification is highly limited due to its slow evolutionary rate. However, the population dynamic of the genotype in Africa seems to be gradually declining. This shows the positive impact of Universal routine vaccination which has been implemented in some African countries, and is being advocated for in others. We recommend the adoption of Molecular surveillance across Africa to further control spread and diversification of Rotavirus.

## Declaration

### Author contribution statement

Babatunde Olanrewaju Motayo: Conceived and designed the experiments; Performed the experiments; Analyzed and interpreted the data; Contributed reagents, materials, analysis tools or data; Wrote the paper.

Olukunle Oluwapamilerin Oluwasemowo: Performed the experiments; Analyzed and interpreted the data; Contributed reagents, materials, analysis tools or data; Wrote the paper.

Babatunde Adebiyi Olusola, Adewale Victor Opayele: Analyzed and interpreted the data; Contributed reagents, materials, analysis tools or data.

Adedayo Omotayo Faneye: Contributed reagents, materials, analysis tools or data.

### Funding statement

This research did not receive any specific grant from funding agencies in the public, commercial, or not-for-profit sectors.

### Competing interest statement

The authors declare no conflict of interest.

### Additional information

No additional information is available for this paper.

## Appendix A Supplementary data

Tempest rota G12 result-1 Tempest rota G12 result-1

supp-table 2 supp-table 2

## References

[1] Troeger C., Khalil I.A., Rao P.C., Cao S., Blacker B.F., Ahmed T. (2018). Rotavirus vaccination and the global burden of rotavirus diarrhea among children younger than 5 years. JAMA. Pediatr..

[2] Ruiz-Palacios G.M., Perez-Schael I., Velazquez F.R., Abate H., Breuer T., Clemens S.C. (2006). Safety and efficacy of an attenuated vaccine against severe rotavirus gastroenteritis. N. Engl. J. Med..

[3] Vesikari T., Matson D.O., Dennehy P., Van Damme P., Santosham M., Rodriguez Z. (2006). Safety and efficacy of a pentavalent human-bovine (WC3) reassortant rotavirus vaccine. N. Engl. J. Med..

[4] Jonesteller C.L., Burnett E., Yen C.J., Tate J.E., Parasha U.D. (2017). Effectiveness of rotavirus vaccination: a systematic review of the first decade of global postlicensure data, 2006-2016. Clin. Infect. Dis..

[5] Estes M.K., Greenberg H.B., Knipe D.M., Howley P.M. (2013).

[6] Bányai K., Kemenesi G., Budinski I., Foldes F., Zana B., Marton S. (2017). Candidate new rotavirus species in Schreiber's bats, Serbia. Infect. Genet. Evol..

[7] Simwaka J.C., Mpabalwani E.M., Seheri M., Peenze I., Monze M., Matapo B. (2018). Diversity of rotavirus strains circulating in children under five years of age who presented with acute gastroenteritis before and after rotavirus vaccine introduction. Vaccine.

[8] Mwenda J.M., Parashar U.D., Cohen A.L., Tate J.E. (2018). Impact of rotavirus vaccines in Sub-Saharan African countries. Vaccine.

[9] Lartey B.L., Damanka S., Dennis F.E., Enweronu C.C., Addo-Yobo E., Ansong D. (2018). Rotavirus strain distribution in Ghana pre- and post- rotavirus vaccine introduction. Vaccine.

[10] Moure U.A.E., Banga-Mingo V., Gody J.C. (2018). Emergence of G12 and G9 rotavirus genotypes in the Central African Republic, january 2014 to february 2016. BMC Res. Notes.

[11] Motayo B.O., Faneye A.O., Adeniji J.A. (2018). Epidemiology of rotavirus a in nigeria: genetic diversity and current insights. J. Pathog..

[12] Japhet M.O., Adesina O.A., Famurewa O., Svensson L., Norgen J. (2012). Molecular epidemiology of rotavirus and norovirus in Ile-Ife, Nigeria: high prevalence of G12P[8] rotavirus strains and detection of a rare norovirus genotype. J. Med. Virol..

[13] Ianiro G., Delogu R., Baba M., Oderinde B.S., Dawurung J., Ruggeri F.M., Fiore L. (2015). Molecular characterization of group A rotavirus strains detected in children with diarrhoea admitted to Nigerian hospitals in 2013. Arch. Virol..

[14] Japhet M.O., Famurewa O., Iturriza-Gomara M., Adesina O.A., Opaleye O.O., Niendorf S. (2018). Group A rotaviruses circulating prior to a national immunization programme in Nigeria: clinical manifestations, high G12P[8] frequency, intra-genotypic divergence of VP4 and VP7. J. Med. Virol..

[15] Matthijnssens J., Heylen E., Zeller M., Rahman M., Lemey P., Van Ranst M. (2010). Phylodynamic analysis of Rotavirus genotypes G9 and G12 underscore their potential for swift global spread. Mol. Biol. Evol..

[16] Rahman M., Matthijnssens J., Yang X., Delbeke T., Arijs I., Taniguchi K. (2007). Evolutionary history and global spread of the emerging G12 human rotaviruses. J. Virol..

[17] Matthijnssens J., De Grazia S., Piessens J., Heylen E., Zeller M., Giammanco G.M. (2011). Multiple reassortment and interspecies transmission events contribute to the diversity of feline, canine and feline/canine-like human group A rotavirus strains. Infect. Genet. Evol..

[18] Rahman M., Matthijnssens J., Saiada F., Hassan Z., Heylen E., Azim T., Van Ranst M. (2010). Complete genomic analysis of a Bangladeshi G1P[8] rotavirus strain detected in 2003 reveals a close evolutionary relationship with contemporary human Wa-like strains. Infect. Genet. Evol..

[19] Suchard M.A., Lemey P., Baele G., Ayres D.L., Drummond A.J., Rambaut A. (2018). Bayesian phylogenetic and phylodynamic data integration using BEAST 1.10. Virus Evol..

[20] Rambaut A., Lam T.T., Max Carvalho L. (2016). Exploring the temporal structure of heterochronous sequences using TempEst (formerly Path-O-Gen). Virus Evol..

[21] Drummond A.J., Nicholls G.K., Rodrigo A.G., Solomon W. (2002). Estimating mutation parameters, population history and genealogy simultaneously from temporally spaced sequence data. Genetics.

[22] Baele G., Suchard M.A., Rambaut A., Lemey P. (2016). Emerging concepts of data integration in pathogen Phylodynamics. Syst. Biol..

[23] Bielejec F., Baele G., Vrancken B. (2016). SpreaD3: interactive visualisation of spatiotemporal history and trait evolutionary processes. Mol. Biol. Evol..

[24] Siddle K., Eromom P., Barnes K.G., Mehta S., JU Oguzie, Odia I. (2018). Genomic analysis of Lassa fever during an increase in cases in Nigeria in 2018. N. Engl. J. Med..

[25] Jenkins G.M., Rambaut A., Pybus O.G., Holmes E.C. (2002). Rates of molecular evolution in RNA viruses: a quantitative phylogenetic analysis. J. Mol. Evol..

[26] Zeller M., Heylen E., Damanka S., Pietsch C., Donato C., Tamura T. (2015). Emerging OP354-like P[8] rotaviruses have rapidly dispersed from asia to other continents. Mol. Biol. Evol..

[27] Seheri M., Nemarude L., Peenze I., Netshifhefhe L., Nyaga M.M., Ngobeni H.G. (2014). Update of rotavirus strains circulating in Africa from 2007 to 2011. Pediatr. Infect. Dis. J..

[28] Nakagomi T., Do L.P., Agbemabiese C.A., Kaneko M., Gauchan P., Doan Y.H. (2017). Whole genome characterization of G12P[6] rotavirus strains possessing two distinct genotype constellations co-circulating in Blantyre, Malawi, 2008. Arch. Virol..

[29] Strydom A., Motanyane L., Nyaga M.M., Joao E.D., Cuamba A., Mandomando I. (2019). Whole genome characterisation of G12 rotavirus strains detected in Mozambique reveals a co-infection with a GXP[14] strain of possible animal origin. J. Gen. Virol..

