# Supplementary material for: Phylogeography and evolutionary analysis of African Rotavirus a genotype G12 reveals district genetic diversification within lineage III
Source: Heliyon. 2019 Oct 21;5(10):e02680. doi: 10.1016/j.heliyon.2019.e02680 (PMC6820252; doi:10.1016/j.heliyon.2019.e02680)
Supplement: Tempest rota G12 result-1 [file mmc1.docx]

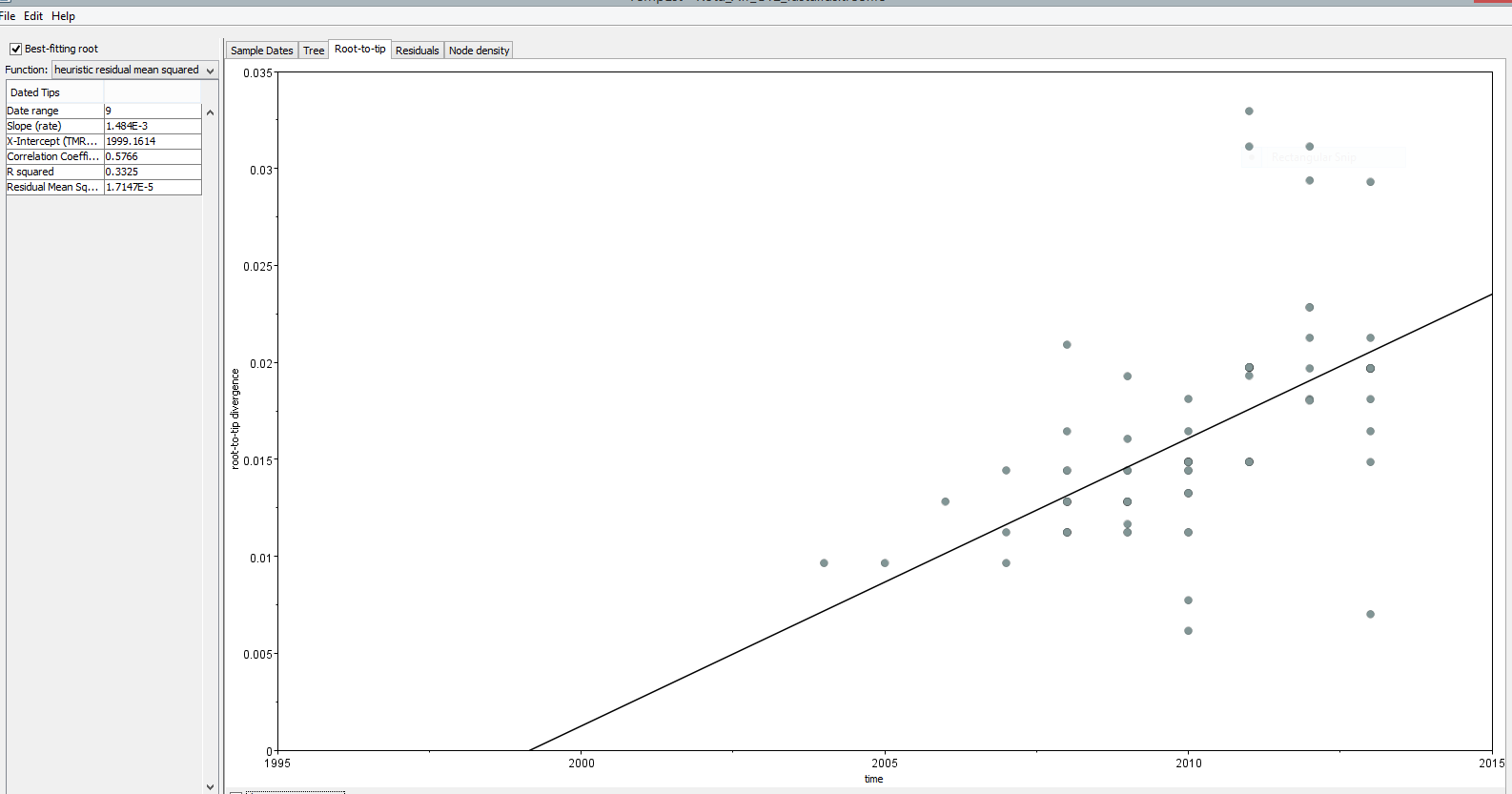


Supplementary Figure 1 shows the chart of the correlation between the genetic divergence and sampling time of the study sequences using TempEst.
